# Twelve quick tips for applying deep learning to animal sounds

**DOI:** 10.1371/journal.pcbi.1014604

**Published:** 2026-08-12

**Authors:** Burooj Ghani, Anne Leonie Baier, Vincent J. Kalkman, Dan Stowell

**Affiliations:** 1 Understanding Evolution Group, Naturalis Biodiversity Center, Leiden, The Netherlands; 2 Biodiversity Hotspots Group, Naturalis Biodiversity Center, Leiden, The Netherlands; 3 Department of Cognitive Science and Artificial Intelligence, Tilburg University, Tilburg, The Netherlands; 4 Leiden Institute of Advanced Computer Science, Leiden University, Leiden, The Netherlands; Montreal, CANADA

## Abstract

Deep learning is transforming the study of animal sound, enabling the automated identification of species, individuals, behaviors, and ecological patterns from large collections of recordings. While bioacoustic machine-learning models are growing more powerful, many biologists—ecologists, behavioral scientists, conservationists—and others working with acoustic data feel unprepared to navigate the computational workflows required to implement them. This article presents practical guidelines covering the full lifecycle of bioacoustic machine learning, including problem definition, data sourcing and annotation, model training, evaluation, deployment, reproducibility, and ethical considerations. Rather than providing a linear checklist, the guidelines outline an iterative framework for building science-led workflows, leveraging transfer learning and open-source tools, evaluating models based on the real-world cost of errors, and addressing domain shift under variable field conditions. Ultimately, this workflow demystifies the software-engineering process, providing a low-barrier and reproducible pathway for researchers applying machine learning to animal sound.

## Introduction

The sounds that animals produce provide insights into behavior, population dynamics, and ecosystems [1–3]. Their study, central to bioacoustics and ecoacoustics, relies on signal processing and related analytical techniques [4,5]. Over the last decade, computational bioacoustics has expanded rapidly, driven by affordable recording technologies and advances in big data, signal processing, and machine learning (ML) [6–8].

Deep learning is transforming computational bioacoustics, enabling analyses of species, behaviors, and acoustic patterns across thousands of hours of recordings [7,9–11]. High-coverage models such as BirdNET, Perch, and NatureLM-audio can now identify thousands of species [12–15]. However, as these tools and studies on dataset design and model generalization proliferate, the path from raw audio to reliable model outputs has become increasingly opaque, with inconsistent evaluation practices [16,17], a still-emerging methodological consensus [7], and substantial unexplained variation in performance across species and regions [18]. In practice, building robust models requires navigating challenges such as domain shift, variable recording conditions, and realistic evaluation frameworks [15,17,19,20]. For researchers without strong computational backgrounds, integrating these elements into a functional workflow remains difficult [21,22].

This article addresses this gap by presenting a practical, step-by-step workflow for ecologists and conservationists adopting ML-based bioacoustics. The growing ecosystem of online tools, cloud computing, public datasets, and open model hubs has made model development more accessible. We distill these resources into 12 practical recommendations [23], building on established guidelines for deep learning in biology [24].

**Fig 1:**
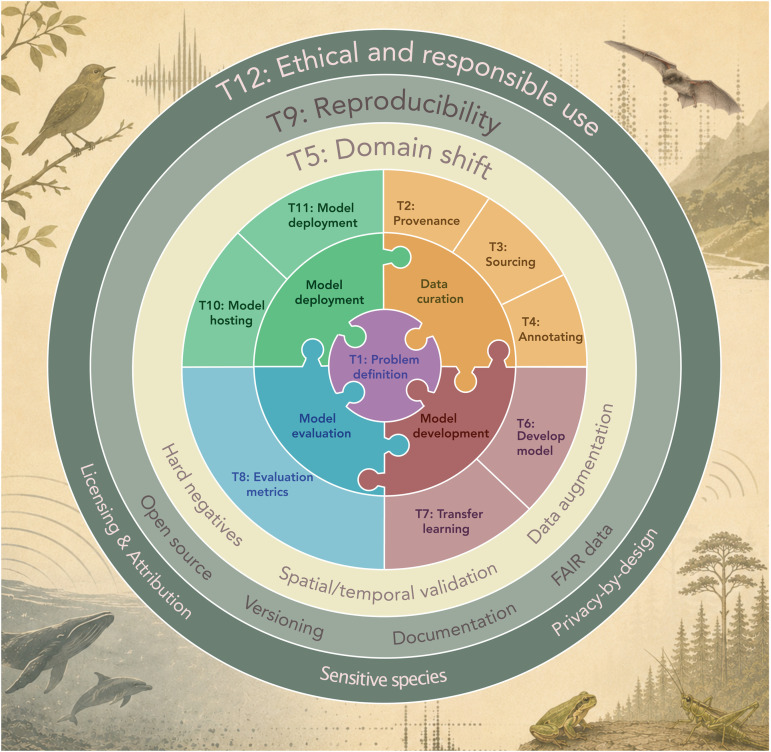
An integrated lifecycle for bioacoustic machine learning. The machine learning research lifecycle is presented as an interconnected, iterative process, emphasizing integration and continuous refinement rather than a linear sequence of steps. At the center, interlocking puzzle pieces illustrate five core stages: Problem definition: aligning ecological questions, deployment demands and technical metrics (*Tip 1*); Data curation: addressing data provenance, sourcing, and annotation (*Tips 2–4*); Model development: applying transfer learning and training principles (*Tips 6–7*); Model evaluation: choosing evaluation metrics (*Tip 8*); and Model deployment: hosting, sharing, and real-world application (*Tips 10–11*). Surrounding these stages, three concentric outer rings represent systemic challenges and cross-cutting principles that must be considered throughout the lifecycle: accounting for domain shift (*Tip 5*), ensuring reproducibility (*Tip 9*), and maintaining ethical and responsible use (*Tip 12*). Figure includes AI-generated components (ChatGPT), modified and finalized by the authors.

These guidelines emphasize reproducibility, data consistency, and collaborative workflows–key principles in modern ecological AI [22,25,26]. The underlying concepts are platform-agnostic and remain stable as interfaces evolve. Although they are also applicable in commercial environments [27,28], this article focuses on implementation using online and open-source tools. This choice aligns with open science principles and ensures that high-level computational monitoring is accessible regardless of institutional funding or local hardware constraints. Consequently, this work provides a low-barrier entry point and a structured workflow from data collection to model deployment (Fig 1).

## Tip 1: Let the science define the pipeline

Start by defining the ecological or biological question before selecting a neural network or building a dataset. *What do you want the deep learning model to achieve?* For example, you may want to **classify** European bat species from echolocation calls, **detect** the presence of a target bird species in a seasonal archive, or use unsupervised learning methods such as **clustering** algorithms to identify recurring patterns across an ecosystem soundscape. Each use case implies different prediction units and resolutions, from clip-level presence–absence to time-resolved events or broader soundscape summaries. This early decision determines how data must be collected and annotated, including the required temporal and spatial resolution.

Beyond the task definition, also account for the intended deployment setting, which constrains acceptable model size, complexity, and inference strategy, from lightweight real-time processing on edge devices to offline analysis of large archival datasets. Together, these task definitions and deployment constraints determine feasible model choices and, consequently, the resulting data design.

## Tip 2: Secure your science with data provenance

Data provenance is the “paper trail” of your research: where audio originates, how it is processed, and how it is annotated. In bioacoustics, recordings become scientifically orphaned without this context; therefore, ensure every file is explicitly linked to sensor settings, GPS location, and timestamps. While general cloud platforms like *Google Drive* [29] and object storage systems like *AWS S3* [30] support collaboration, they lack robust versioning and persistent identifiers required for reproducibility [31]. Instead, use scientific data repositories: Platforms such as *Hugging Face* [32] additionally support dataset versioning and structured access, while services like *Zenodo* [33] provide persistent identifiers (DOIs) for long-term archiving and citation. For richer metadata structures, use domain-specific systems such as *Tethys* [34], which support spatio-temporal tracking and integration of acoustic data with environmental covariates.

Recording parameters—microphone type, sampling rate, bit depth, gain settings, and recording schedules—strongly affect both signal properties and model performance. For example, models trained on 48 kHz recordings from specialized sensors may not generalize to 22 kHz edge-device data. Document all configurations using standardized formats such as the GUANO metadata standard [35] for file-level tags, or emerging exchange standards like the acoustic adaptation of Camtrap DP [36]. Finally, adopt FAIR principles (Findable, Accessible, Interoperable, Reusable) and use broader ecological standards such as EML (Ecological Metadata Language) [37] or Darwin Core [38] to ensure integration with global biodiversity databases, such as GBIF. This transforms your local project into a contribution to global conservation science.

## Tip 3: Source your data with intent

High-quality training data is decisive for bioacoustic deep learning, but building a dataset from scratch is rarely efficient. Instead, adopt a three-pronged strategy that balances speed, scale, and specificity, combining curated datasets for prototyping, repositories for scaling, and local recordings for validation.

First, **curated, ML–ready datasets** provide an efficient starting point and improve reproducibility. Platforms such as *Zenodo* [33] and *Hugging Face* [32] host datasets with validated labels, standardized formats, and predefined splits, reducing preprocessing effort. Benchmark datasets such as BEANS [39], Birb [40], BirdSet [41], and InsectSet459 [42] support transparent model comparison through fixed tasks and evaluation protocols. Aggregators such as *Datasets for Bioacoustics* [43] help locate datasets tailored to specific taxa, environments, or tasks.

**Fig 2:**
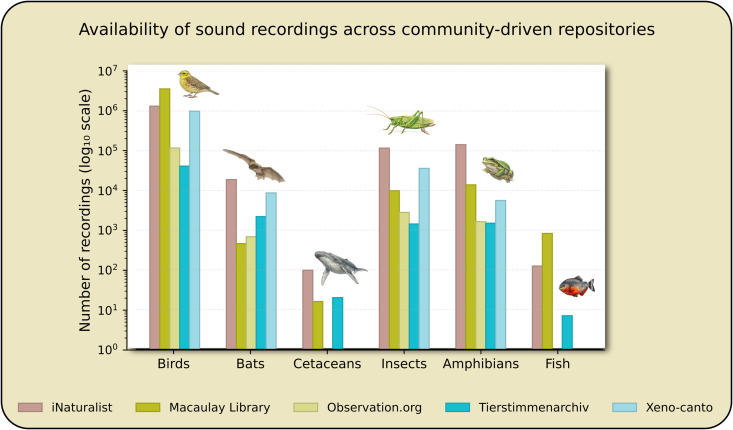
Number of sound recordings per taxonomic group across major community-driven repositories, illustrating repository strengths and gaps. Counts represent global totals and are approximate due to differences in search functionality and taxonomic classifications across portals; high global totals do not necessarily imply coverage in all geographic regions. Data current as of June 2026. Figure includes AI-generated components (ChatGPT), modified and finalized by the authors.

Second, **large community-driven repositorie**s provide the taxonomic and geographic breadth needed for generalizable models. Platforms including *Xeno-Canto* [44], *Macaulay Library* [45], *iNaturalist* [46], *Observation.org* [47], and the *Tierstimmenarchiv* [48] host millions of recordings that can be filtered by species, location, or quality (Fig 2). Broader aggregators, most notably *GBIF* [49] offer unified access across sources and enable DOI assignment for reproducible data queries [50].

Third, use **targeted local recordings** to ground models in their deployment environment. While large repositories provide scale, their focal recordings are often cleaner and may not reflect the acoustic complexity of passive monitoring, leading to domain shift (see Tip 5). Incorporate local data into training via **transfer learning** (see Tip 7). This lightweight adaptation strategy requires relatively little data and helps calibrate models to site-specific hardware and noise conditions.

## Tip 4: Make annotation a team effort

Even when using existing ML-ready datasets for training (see Tip 3), creating reliable annotations remains essential for validation and fine-tuning. Bioacoustic recordings often contain overlapping vocalizations and ambiguous signals making consistent labeling challenging. Establish clear labeling protocols including standardized confidence scales and explicit rules for handling “acoustic clutter.” Ensure annotators consistently label background noise and non-target species to provide the “hard negatives” required for robust model performance (see Tip 5).

Define the appropriate level of annotation detail early, as this determines both workload and model capability. **Weak labels** indicate species presence within a recording and scale well to large datasets, whereas **strong labels** mark the exact timing of vocalizations, enabling fine-grained evaluation at substantially higher annotation cost. Because weak labels can be misleading in complex soundscapes, many projects adopt a hybrid approach, combining small, strongly labeled subsets for ground truth with larger weakly labeled datasets. Some repositories now support this workflow; for example, *Xeno-Canto* [44] allows sharing of strong annotations alongside recordings.

Use appropriate tools to support collaborative annotation. Desktop applications such as *Audacity* [51], *Raven* [52], *Sonic Visualiser* [53], and *AviaNZ* [54] remain essential for detailed spectrogram inspection while browser-based tools such as *Spectrolipi* [55] and *SignaVis* [56] provide quick accessibility. For large-scale projects, web-based platforms (e.g., *APLOSE* [57], *NEAL* [58]), *Whombat* [59], *ARBIMON* [60], *WildTrax* [61]) enable multi-user workflows within centralized databases. Efficiency can be further improved through human-in-the-loop active learning [62] and hybrid workflows [63] that integrate specialist review with non-expert crowdsourcing (see Tip 7).

Implement formal **quality control** to maintain annotation integrity. Use shared guidelines and reference clips, and regularly assess Inter-Annotator Agreement (IAA) [64] to quantify consistency. For ambiguous cases, apply a consensus or referee workflow [65] in which experts resolve disagreements. Finally, track annotation changes using version control (e.g., Git, database logs, or revision tracking) to ensure transparency and long-term reproducibility [31].

## Tip 5: Bridge the gap from repository to reality

A central challenge in bioacoustic machine learning is **domain shift**: models trained on curated recordings (e.g., crowd-sourced repositories) often fail to generalize to real-world deployment conditions [66,67]. Differences in recording context (focal recordings vs. passive acoustic monitoring), acoustic environment, hardware, geography, and temporal variation can all reduce predictive performance [17,39,41]. This mismatch arises because high-quality focal recordings rarely reflect the “messier” reality of passive acoustic monitoring (PAM). Although domain shift cannot be fully eliminated, its effects can be mitigated through targeted dataset design and evaluation strategies. Therefore, ensure that evaluation data reflects deployment conditions rather than just more focal data.

To improve robustness, avoid “positive-only” datasets. Because ecological recordings are background-dominated, models must also learn what *not* to detect. Include explicit **negative examples** by incorporating “noise” such as wind, rain, and non-target species, ensuring these examples resemble the acoustic characteristics of target classes. Include such “hard negatives” to reduce false positives and improve discrimination between biological signals and environmental noise. Use **data augmentation** (e.g., noise overlay, pitch shifting, time stretching, mixup) to expose models to realistic variability. When possible, use an iterative **transfer learning** approach (see Tip 7) by training lightweight classifiers on pretrained embeddings (e.g., BirdNET or Perch [12,13]) with small, representative local datasets.

Crucially, ensure that evaluation protocols reflect real-world variability [17,19]. Avoid purely random dataset splits, as they are prone to site- or time-based leakage. Be cautious when constructing test sets from public archives, as they may overlap with training data used for major pretrained models, effectively contaminating the test set and inflating performance estimates. Instead, use **spatial or temporal block cross-validation**, testing models on sites or time periods not seen during training. Without this, models may overfit to site-specific background noise rather than learning generalizable species characteristics.

## Tip 6: Develop your model smartly

Model development is the stage in which candidate approaches are tested, refined, and evaluated, whether by fine-tuning a pre-trained network, fitting a lightweight classifier on embeddings, or training a model more extensively on labeled audio data, all while adhering to established good practices [7,68]. In many contemporary bioacoustic workflows, model development centers on adapting pretrained models or embeddings rather than training models entirely from scratch (see Tip 7).

Treat model development as an iterative process rather than a single step. Begin with small, “shallow” pilot experiments on subsets of your data to identify dataset biases, site-based leakage (see Tip 5), unstable learning behavior, or generalization failures before committing significant time or resources to full-scale training.

The computational demands of model development can vary substantially depending on the chosen approach, so the choice of training environment—local or remote—should reflect dataset size, available resources, and the intended use of the model.

Choose your training environment—local or remote—based on the iterative workflow. Use **local setups** when full control over data, reproducibility, and computational configuration are critical, but rely on **cloud platforms or high-performance computing (HPC)** resources to scale experiments. Browser platforms such as *Google Colab* [69], *Kaggle Notebooks* [70], or *AWS SageMaker Studio Lab* [71] enable rapid prototyping without specialized hardware, lowering the barrier to entry—despite potential costs or data transfer limitations.

Start simple, evaluate often, and scale computational resources only once a workflow has proven capable of generalizing reliably.

## Tip 7: Use transfer learning over training from scratch

Before investing significant time and computational resources in model development, consider whether existing pre-trained models or learned feature representations can be adapted for your application. In many bioacoustic applications, this means that model training consists not of building a network from scratch, but of fine-tuning a pre-trained model or fitting a lightweight classifier on top of precomputed embeddings. **Transfer learning**, which reuses feature representations (**embeddings**) from previously trained models, is now a central strategy in computational bioacoustics [19]. Instead of assembling large datasets to train from scratch, collect smaller, targeted datasets that reflect deployment conditions and adapt pre-trained **foundation models** [13,66]. This approach is particularly useful when labeled data are limited or costly [20]. By leveraging embeddings learned from large and diverse audio collections, models can capture general acoustic structure that transfers across species, call types, and taxa, enabling a wide range of downstream tasks without full retraining [13,39,72].

Do not assume pre-trained models will generalize automatically. Guide transfer learning by the ecological question and deployment conditions defined at the outset (see Tip 1). Mismatches in species composition, recording conditions, or regional soundscapes (e.g., high-frequency insect noise absent from the original training data) can limit transferability and result in poor generalization [18].

Use dedicated platforms to streamline adaptation. Frameworks such as *BacPipe* [21], *OpenSoundscape* [73], and *avex* [74] support building task-specific classifiers on precomputed embeddings and comparing model performance. Taxon-specific tools such as *Bird Sounds Global (BSG) model builder* [75] and *BSG-BATS* [76] enable fine-tuning on locally annotated soundscapes, while the *Hugging Face Hub* [32] supports reuse and sharing of bioacoustic models. Model-specific resources such as *Perch* [13], *BirdNET-Analyzer* [12], *AvesEcho* [19] and *ANIMAL-SPOT* [77] provide pretrained models and tooling for transfer across taxa.

Transfer learning also enables more iterative “agile modeling” workflows [78,79]. Rather than exhaustively annotating large datasets upfront, experts can begin with a small set of labeled examples and use embedding similarity search to rapidly identify related sounds within large unlabeled collections. Human-in-the-loop active learning can then refine lightweight classifiers by prioritizing ambiguous or informative examples (“hard negatives”) for bioacoustic labeling workflows [80–82]. Because embeddings only need to be computed once, these workflows are computationally efficient and enable robust task-specific models to be developed from relatively small labeled datasets [19,79].

## Tip 8: Select metrics based on the cost of error

Evaluation metrics should not be treated as default settings but should reflect the ecological consequences of model errors [16]. In rare-species monitoring where missed detections are unacceptable, prioritize **recall**, even at the cost of increased false positives requiring manual review. Conversely, in automated alert systems where false positives trigger costly downstream actions, prioritize **precision**.

To evaluate a model’s ability to rank detections independent of a fixed decision threshold (i.e., the confidence cutoff used to assign presence), use threshold-free measures like **AUROC** (area under the curve) or **mAP** (mean average precision) [17,19]. This ranking-based evaluation is particularly useful in multi-label tasks.

When reporting ranking metrics, account for the strong class imbalance typical of bioacoustic datasets, where some classes have far more recordings than others. Use **class-balanced** formulations (e.g., macro-averaged precision, macro-averaged recall, or class-wise mAP) [83] to ensure that rare species contribute equally to performance estimates and to allow decision thresholds to be tuned per species or deployment context without misrepresenting overall model skill [84]. Similarly, use balanced accuracy rather than standard accuracy when class frequencies differ substantially.

## Tip 9: Use libraries for reproducibility

Open-source Python libraries form the computational backbone of bioacoustic model development [31]. Rather than implementing functionality from scratch, use established libraries to reduce development time, minimize errors, and promote standardized, reproducible pipelines. Reproducibility is a guiding principle that should be maintained throughout the machine learning lifecycle; here, we highlight widely adopted libraries as practical tools for achieving this goal. These libraries are not confined to a single stage of the workflow, but instead provide cross-cutting infrastructure for data access, preprocessing, augmentation, training, evaluation, and deployment.

A key starting point is the usage of libraries for data access and metadata retrieval. As projects increasingly involve thousands of recordings, data collection should also move beyond manual downloads. Utilize Application Programming Interfaces (APIs) for structured queries and bulk downloads of audio files and metadata (often referred to as *scraping*). Python wrappers such as xenocanto-api [85] and pyinaturalist [86] simplify programmatic access and integration into preprocessing pipelines.

Once data access is established, dedicated libraries can be used to process recordings through automated and standardized pipelines to ensure consistency between training and inference. Libraries such as Torchaudio [87] and librosa [88] support audio input/output, preprocessing, and spectrogram generation. Audiomentations [89] provides efficient data augmentation methods (e.g., noise addition, pitch shifting, time stretching; see Tip 5). Frameworks including Pytorch [90], pytorch-lightning [91], tensorflow [92] enable efficient model development and training without reimplementing low-level functionality. High-level libraries such as https://pypi.org/project/opensoundscape/ OpenSoundscape [73] further streamline these workflows by integrating preprocessing, labeling, and training into reproducible pipelines.

Use deployment-oriented frameworks when models must run on edge devices or in end-to-end monitoring systems. For example, deployment-oriented frameworks such as https://github.com/acoupi/acoupi acoupi [93] support the integration of bioacoustic classifiers on edge devices such as Raspberry Pi, providing end-to-end workflows spanning recording, processing, detection, and data management.

Using well-established libraries improves comparability across studies by relying on shared implementations, allowing researchers to focus on experimental design and ecological interpretation rather than low-level engineering. Although they streamline development, libraries should not be treated as black boxes; understanding key assumptions and defaults (such as spectrogram FFT settings) remains essential for reliable and interpretable results.

## Tip 10: Make your models reusable, not just public

Once a model is developed, make it accessible online in a form that supports reuse, collaboration, and reproducibility. Share not only model weights but also code, preprocessing pipelines, and training configurations so others can reproduce results, apply the model in a consistent setting, and adapt it to new ecological questions. This level of transparency is essential for trustworthy and reproducible ML workflows [26,94] —a published model is not automatically a usable one. Ensure usability by providing complete documentation. Include preprocessing steps, example inputs, and clear instructions for running inference on new data so results can be replicated across environments.

Use appropriate platforms to support sharing and reproducibility. Host code, checkpoints, and scripts on version-controlled repositories such as *GitHub* [95], *GitLab* [96], and *Codeberg* [97]. Share models and interactive demos via *Hugging Face* [32], which also supports fine-tuning and deployment through the broader Hugging Face ecosystem. Use container platforms such as *Docker Hub* [98] to distribute fully reproducible environments, and archive releases on *Zenodo* [33] to assign persistent DOIs for citation.

Well-documented models enable downstream reuse, allowing others to extend analyses or integrate them into new studies. Without clear documentation and reproducible workflows, however, a public model remains difficult to use in practice.

## Tip 11: Make deployment part of your workflow

Training and sharing a model is only the first step—ensure it is deployable. A reusable model package is not necessarily a deployable system: deployment requires that the model run reliably on new recordings under real-world computational and environmental constraints. Plan for deployment conditions early, as they often differ from training settings and impose constraints such as limited compute, continuous data streams, low-power edge devices, and noisy environments. Deployment bridges the gap between a repository model and a system that performs reliably in real-world workflows [22,73].

Pick a deployment strategy that matches your use case. Choose local deployment for small- to medium-scale analyses or when full control is required. Many pre-trained models (e.g., *BirdNET-Analyzer* [12], *AvesEcho* [19], *Perch* [13,99]) or notebooks adapted from *Hugging Face* [32] repositories provide scripts for batch processing and automated inference, enabling users to run models on their own hardware.

For users with the necessary technical expertise, a more advanced option for constrained scenarios is to deploy models directly on low-power edge devices (e.g., embedded field sensors), where strict limits on model size, power consumption, and latency may require simplified architectures or optimized pipelines.

Use online deployment for accessibility and scale. Run models remotely on web-based platforms such as *Hugging Face Spaces* [32] and *ARISE* [100] to allow users to upload audio and run inference without managing infrastructure.

Maintain strict consistency between training and inference. Match preprocessing steps (e.g., sampling rate, normalization, feature extraction), as small mismatches can significantly degrade performance. When needed, optimize deployment with batch processing, appropriate hardware (CPU vs GPU), and specialized pipelines for ultrasonic recordings. Finally, evaluate models under realistic deployment conditions. Performance on curated datasets often overestimates real-world performance, so test models in the environments where they will be used [17,18].

## Tip 12: Protect species and people

Treat bioacoustic data and models as ethically and legally sensitive throughout the project lifecycle. When sourcing data, follow licensing agreements (e.g., *Creative Commons* [101]) and ensure proper attribution of community-contributed recordings. Prioritize open data and standardized attribution to support equity, transparency, and collaboration in research [102].

Balance data sharing with ecological risk. Avoid publishing precise GPS coordinates for sensitive or endangered species, as this may facilitate poaching or disturbance. When necessary, obscure spatial data or restrict access through secure, vetted platforms.

Account for privacy during deployment. Acoustic monitoring can capture human activity, raising surveillance concerns. Mitigate this by implementing privacy-by-design approaches, e.g., with *acoupi* [93], which enables on-device processing with selective storage or deletion of raw audio after detection. However, additional components (e.g., speech or voice-activity filtering) may be required when human privacy is a concern.

Additional measures, such as engaging with local communities, can further support transparency and trust. Apply these practices consistently to ensure that bioacoustic AI supports conservation goals without compromising human privacy or species safety.

## Conclusions

Modern online infrastructures have democratized bioacoustic machine learning, making it more accessible than ever. By following these 12 tips, researchers can navigate the full workflow—from defining an ecological question to deploying a functional model.

As illustrated in Fig 1, this workflow is not linear but iterative and interconnected. It begins with planning, where research questions define scale and resolution; continues through data engineering, where collection and annotation establish a robust foundation; and advances to technical development, where transfer learning and efficient training adapt models to local conditions. It culminates in deployment, where models are tested under real-world constraints and ethical considerations.

Across all stages, challenges such as domain shift and data provenance must remain central. Model success should not be judged by high accuracy on curated datasets, but by the ability to deliver reliable, meaningful insights in noisy and variable field conditions.

This article provides a practical guide to these workflows, while readers seeking deeper technical detail on deep learning for computational bioacoustics are referred to Stowell [7]. Although tools and platforms will continue to evolve, the underlying principles of careful experimental design and ecological insight remain constant. By combining accessible infrastructure with open and responsible practices, researchers can build transparent, reproducible, and impactful bioacoustic systems that directly support biodiversity monitoring and conservation.
